# Computationally Modelling Cholesterol Metabolism and Atherosclerosis

**DOI:** 10.3390/biology12081133

**Published:** 2023-08-14

**Authors:** Callum Davies, Amy E. Morgan, Mark T. Mc Auley

**Affiliations:** 1 Department of Physical, Mathematical and Engineering Sciences, University of Chester, Chester CH1 4BJ, UK; caldavies000@gmail.com; 2 School of Health & Sport Sciences, Liverpool Hope University, Liverpool L16 9JD, UK; morgana1@hope.ac.uk

**Keywords:** atherosclerosis, cholesterol, low density lipoprotein cholesterol (LDL-C), plaque, cardiovascular disease (CVD), mathematical model

## Abstract

**Simple Summary:**

Heart disease and stroke are major global health problems. There are many risk factors for these conditions. However, the main risk factor is high levels of low-density lipoprotein cholesterol (LDL-C). LDL-C is involved in the formation of plaques which eventually lead to either a heart attack or a stroke. The biology associated with this process is exceptionally complex. Computational modelling can be used to understand this complexity. In this work computational modelling was used to better understand the relationship between high levels of LDL-C and plaque progression. The model was able to identify therapeutic interventions which are effective at slowing plaque growth.

**Abstract:**

Cardiovascular disease (CVD) is the leading cause of death globally. The underlying pathological driver of CVD is atherosclerosis. The primary risk factor for atherosclerosis is elevated low-density lipoprotein cholesterol (LDL-C). Dysregulation of cholesterol metabolism is synonymous with a rise in LDL-C. Due to the complexity of cholesterol metabolism and atherosclerosis mathematical models are routinely used to explore their non-trivial dynamics. Mathematical modelling has generated a wealth of useful biological insights, which have deepened our understanding of these processes. To date however, no model has been developed which fully captures how whole-body cholesterol metabolism intersects with atherosclerosis. The main reason for this is one of scale. Whole body cholesterol metabolism is defined by macroscale physiological processes, while atherosclerosis operates mainly at a microscale. This work describes how a model of cholesterol metabolism was combined with a model of atherosclerotic plaque formation. This new model is capable of reproducing the output from its parent models. Using the new model, we demonstrate how this system can be utilized to identify interventions that lower LDL-C and abrogate plaque formation.

## 1. Introduction

Cardiovascular disease (CVD) is the leading cause of death globally [1,2,3,4,5]. Many risk factors are associated with CVD. Non-exhaustively, this includes; physical inactivity [6], genetic predisposition [7], obesity [8], metabolic syndrome [9], nutrition [10,11], tobacco smoke [12], gut microbiome changes [13], epigenetic changes [14], and elevated homocysteine [15]. However, among the risk factors for CVD, elevated low-density lipoprotein cholesterol (LDL-C) remains the gold standard predictor of CVD risk [16]. The pathophysiological explanation for this is that LDL-C is pivotal to the aetiology of atherosclerotic CVD (ASCVD). Atherosclerosis is a progressive disease which is underpinned by a chronic inflammatory response [17]. The initial step in this process involves LDL penetrating the arterial endothelium and entering the intima. Lipid laded foam cells are then generated as a consequence of macrophages engulfing the LDL [18]. Foam cells generate fatty streaks which mature into plaques [19]. These plaques narrow the arterial lumen, resulting in reduced blood flow [20]. If a plaque ruptures it can lead to a stroke or a heart attack; diseases which are the principle clinical manifestations of ASCVD [21]. Thus, a medical imperative exists to identify therapeutic avenues which lower ASCVD risk. Existing pharmacological agents target various aspects of cholesterol metabolism in order to lower LDL-C and decrease CVD risk [22,23,24]. However, these therapeutics are not universally effective [25,26,27]. As a result, a continual drive exists to identify novel modalities which are capable of lowering LDL-C [28,29].

To identify suitable ways of lowering LDL-C, it is necessary to understand the regulation of whole-body cholesterol metabolism [30]. Whole body cholesterol metabolism is an inherently complex multicomponent system, and its behaviour is maintained by an array of mechanisms interacting in a coordinated fashion [31,32]. The mechanisms which regulate whole-body cholesterol metabolism are subject to interindividual variability due to both lifestyle choices and genetic heterogeneity [33]. This means an individuals’ LDL-C level can be influenced by their diet [34], differences in cholesterol absorption [35], variations in endogenous sterol synthesis [36], and drug response [37]. Attempting to understand the complex nature of whole-body cholesterol metabolism and atherosclerosis is further exacerbated by ageing. Ageing is associated with the dysregulation of this system [8,33,38,39,40]. Increasing age closely corelates with a rise in LDL-C in both males and females; a phenomenon which has been observed across various population groups. Indeed, age is the most significant risk factor for ASCVD [41,42].

ASCVD progression is influenced by a variety of other processes. These include hemodynamic mechanisms such as arterial wall shear stress [43,44]. Other variables which contribute to plaque development include reactive oxygen species [45], apoptosis [46], vascular proliferation [47], matrix degradation and inflammaging [48]. Reductionist experimental approaches have in the main been used to deal with this complexity [49]. Such meticulous empirical work has contributed significantly to advancing the understanding of cholesterol metabolism and ASCVD. However, reductionist approaches are an insufficient way of gaining a complete understanding of any biological system. When cholesterol metabolism and atherosclerosis are viewed through a systems biology lens, they can be represented as a mathematical model, which is defined by a series of complex overlapping biological networks [50,51]. When such a representation is dynamically simulated this generates a more integrated interpretation of the biology which can lead to novel insights [52].

Various aspects of cholesterol metabolism/atherosclerosis have been modelled previously [51,53]. Existing work is categorised into several areas; this includes models of cholesterol biosynthesis/the mevalonate pathway [54,55], whole-body cholesterol metabolism [56,57], intracellular cholesterol homeostasis [58], reverse cholesterol transport [59], lipoprotein processing [60], LDL receptor mediated endocytosis [61], and haemodynamic/multi-physics/pathophysiological models of atherosclerosis [62,63]. These models have generated a wealth of insights which have deepened the understanding of cholesterol metabolism/atherosclerosis [53,64]. To date however, no model has been developed which fully captures how whole-body cholesterol metabolism intersects with atherosclerosis. The main reason for this is one of scale. Whole body cholesterol metabolism is defined by macroscale physiological processes, while atherosclerosis operates mainly at the microscale. The aim of this work is to address this theoretical gap by combining a model of cholesterol metabolism with a model of atherosclerosis. To do this, two existing models were joined together to create a more complete representation of the nexus between cholesterol metabolism and atherosclerosis. The combined model is capable of reproducing output from its parent models. The new model is also used to conduct several in silico experiments. Findings from the experiments broadly align with previous published experimental data and suggest that pharmacological interventions can have a significant impact on LDL and plaque size. Furthermore, the work illustrates the importance of diet in tandem with pharmacological intervention in patients with hypercholesterolaemia.

## 2. Materials and Methods

### 2.1. Model Selection

After conducting an extensive literature review [53], a substantial number of models were identified which represent various aspects of cholesterol metabolism/atherosclerosis. Based on this survey, it was concluded that pre-existing models could be used for this work, and that a bespoke model was unnecessary. A key criterion for selection was that model code needed to be publicly available. To further increase experimental rigour, model output had to have been validated. A way to ensure validation is to source the model from BioModels (https://www.ebi.ac.uk/biomodels/, accessed on 14 May 2023) [65]. BioModels is a database of models encoded in systems biology markup language (SBML) [66,67]. SBML is the leading model exchange format used in systems biology. BioModels is subdivided into curated and non-curated sections. Non-curated models may have valid SBML but are awaiting curation. A curated model has been fully validated. Put simply, it has been published in a peer-reviewed journal, and its output has been verified by BioModels curators.

BioModels contained one validated model of whole-body cholesterol metabolism by Mc Auley et al. (2012) (BIOMD0000000434) [56]. The model has a simple topology which includes the major processes that represent cholesterol balance in the human body. The model has been extensively used to investigate cholesterol metabolism [57,68,69,70,71]. This recapitulated and underscored its utility for this work. BioModels has eight non-curated models of atherosclerosis. There are no curated models. Two of the eight non-curated models focus on atherosclerosis pathophysiology, specifically atherogenesis [72], and atheroma formation [73]. Because both models capture the underlying pathophysiology of atherosclerosis, their SBML was imported into the model development and simulation software tool COPASI (version 4.30) [74]. After they were examined, the atherogenesis model [72] (MODEL1002160000) was selected. The rationale for choosing this model was that its seventeen-reaction structure precisely matched its description in its corresponding publication. Moreover, when simulations were conducted in COPASI, model output aligned with peer-reviewed published results. This provided confidence in model validity. Another reason for its selection is that there is commonality with the cholesterol model that was selected.

The model possesses a simple topology and a logical schema which captures the key mechanisms underpinning atherosclerotic plaque formation. In addition, both models are composed of ordinary differential equations (ODEs), which facilitated model merging.

### 2.2. Creating a Unified Network Diagram

**Figure 1:**
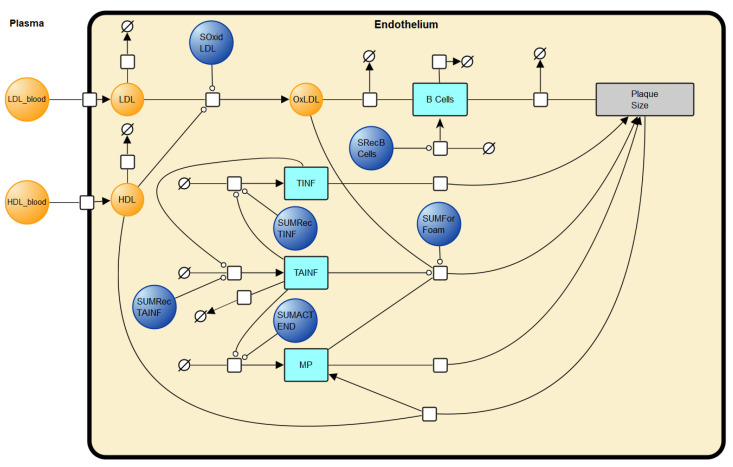
SBGN diagram of the Gomez-Cabrero et al. (2011) [72] model of atherogenesis.

**Figure 2:**
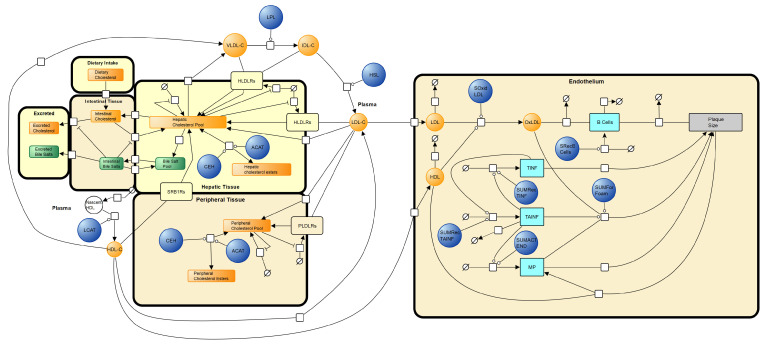
Combined SBGN network diagram of whole-body cholesterol metabolism and its intersection with atherosclerosis. Adapted from [56,72].

The first step in model merging was to identify suitable intersection points. This was undertaken by examining model network diagrams. The Mc Auley et al. (2012) model is represented in systems biology graphical notation (SBGN) [75,76]. See Figure S1, Supplementary File S1. The Gomez-Cabrero et al. (2011) [72] model has a Biological Pathway Exchange Model (BioPAX) associated with it [77]. To visualise the BioPAX file in SBGN, the BioPAX file was imported in to Vanted (Version 2.8.1), an SBGN visualisation and analysis software tool [78]. When visualised in Vanted, the diagram did not describe the model reactions completely (Figure S2, Supplementary File S1). To rectify this, Vanted was used to update the network diagram (Figure 1). To do this, the model SBML was imported into COPASI, and the reaction list identified. The reaction list was utilised to add more detail to the SBGN diagram. The updated Gomez-Cabrero et al. (2011) [72] diagram was then merged with the SBGN of the Mc Auley et al. (2012) [56] model to create a unified network diagram. The logical points of intersection were the HDL (high density liprotein)_blood and LDL_blood species (variables). These species refer to the concentration of LDL-C and HDL-C in the blood. These are the same species as ‘LDLC’ and ‘HDLC’ in the Mc Auley et al. (2012) [56] model. These species were used to join the two models in SBGN. The new network diagram is outlined in Figure 2. 

### 2.3. Merging the SBML Files

The SBML files for both models were imported into COPASI. This was done by using the import SBML function, followed by the ‘add to model’ feature to load the second model. At this stage, both models were located within the same file but behaved independently. Before merging the models, some technical issues needed resolving. Firstly, it was necessary to verify that the models entered a steady state. The Gomez-Cabrero et al. (2011) [72] model entered an asymptotically stable steady state. The Mc Auley et al. (2012) [56] model did not enter a steady state. However, once the concentrations of the following sink species, EC (excreted cholesterol), HLDRLD (hepatic LDL receptor degradation), PLRLRD (peripheral LDL receptor degradation), PSS (peripheral steroid synthesis), and EBS (excreted bile salts) were fixed, the model entered a steady state.

Next, the time setting of both models was aligned. The Mc Auley et al. (2012) [56] model is set to days, whereas the Gomez-Cabrero et al. (2011) [72] atherogenesis model is in weeks. All time dependent reactions and quantities, from this model, were converted to days. All time dependent parameters for this model are listed as global quantities, and the conversions can be found in Table S1, Supplementary File S1.

The models were coupled at two overlapping points (LDLC and HDLC). In the Gomez-Cabrero et al. (2011) [72] work, LDL and HDL are represented by LDL_blood and HDL_blood respectively. In the Mc Auley et al. (2012) [56] model, these are represented by LDLC and HDLC. The species LDL_blood and HDL_blood were deleted, and the reactions edited to join the models. Following this, the global quantity ‘M’ was created; M serves as an implicit conversion constant to convert LDLC with units dL/mg to dimensionless LDL_blood. This simple conversion is described in Equations (1) and (2):

(1) $$LDL_{blood}=LDLC*M$$

(2) $$HDL_{blood}=HDLC*M$$

LDL is a state variable described as the proportion of LDL-C in the intima with respect to LDL-C in the blood [72]. M is important to not only facilitate the conversion from concentration to a dimensionless state variable, but also to implicitly convert the scale from whole-body to the cellular process of atherosclerosis. The value of M, was arbitrarily set to 1 initially. To complete the reaction change, new rate laws were required as the existing mass action (irreversible) rate laws only used two parameters which were KinLDL and LDL_blood or KinHDL and HDL_blood. New functions with the name ‘Rate law for InHDL’ and ‘Rate law for InLDL’ were created. The formula for the InLDL rate law was set as KinLDL∗LDLC∗M. The formula for the InHDL rate law was set as KinHDL∗HDLC∗M.

Lastly, the compartment representing the endothelium, in the Gomez-Cabrero et al. (2011) [72] model, was updated from ‘compartment’ to ‘endothelium’ to better describe the new model. Initial concentrations of species and reactions list for the merged model are found in Tables S2 and S3, Supplementary File S1. The ODEs can be found in Supplementary File S2.

### 2.4. Reparameterization

A parameter estimation for two key components of the merged model, M and DC (dietary cholesterol), was conducted. To do this, a simulation over 700 days was run in the Gomez-Cabrero et al. (2011) [72] model. The output was saved as a text file and uploaded into the merged file as experimental data for parameter estimation. There, PLAQUE was selected as dependent. The parameter estimation identified a DC value of 1050.92 mg/day (rounded to 1051 mg/day), and an M value of 0.0155561 as the optimal parameter values to reproduce the experimental data. This is a significant amount of cholesterol to be ingested; although it is biologically possible to consume cholesterol levels this high, it would be very unusual. This results in elevated LDL-C, however, this is similar to the Gomez-Cabrero et al. (2011) [72] model, where the LDL-C regime is defined as ‘high’.

**Figure 3:**
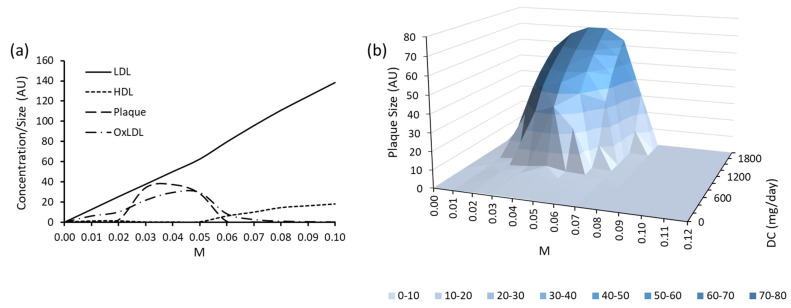
Parameter scan results of (**a**) M on LDL, HDL, plaque size and oxLDL and (**b**) M and DC in tandem on plaque size. For (**a**), the solid black line (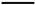) represents LDL, the square dot line (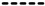) signifies HDL, the long dash line (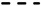) is representative of plaque size, and the long dash dot line (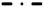) characterises oxLDL.

To investigate the impact of M further, a parameter scan was conducted between the values of 0.01 and 0.1, with interval sizes of 0.01. The behaviour of LDL, HDL, plaque size, and oxidised LDL (oxLDL) was examined (Figure 3a). Increasing M raised the amount of LDL entering the endothelium, with a peak plaque size observed between values of 0.02 and 0.07. To examine the effect of M on plaque size more closely, the parameter M was investigated in tandem with DC (Figure 3b). To do this, the two parameters of interest were scanned simultaneously using the parameter scan tool in COPASI. DC was scanned between the values of 0–2000 mg/day using an interval size of 200 mg/day (11 values), and M was scanned simultaneously between 0–0.15 using an interval size of 0.01 (16 values). In total 176 data points were created. The simulations were run for 700 days. Data was extracted from COPASI to Microsoft Excel version 1808 and a 3D surface map created using the 176 data points; gaps between these points were interpolated as standard to the software. Increased DC was associated with an increase in plaque size. However, plaque size was greatest when M was between 0.04–0.05. DC had a nominal effect on plaque size when M was greater than 0.08. Furthermore, when DC was <600 mg/day, M had little impact on the plaque size. The merged model, with these identified parameter values, was found to be asymptotically stable at a resolution of 1 × 10^−8^ (Supplementary File S3).

## 3. Results

### 3.1. Comparing the Merged System with the Parent Models

**Figure 4:**
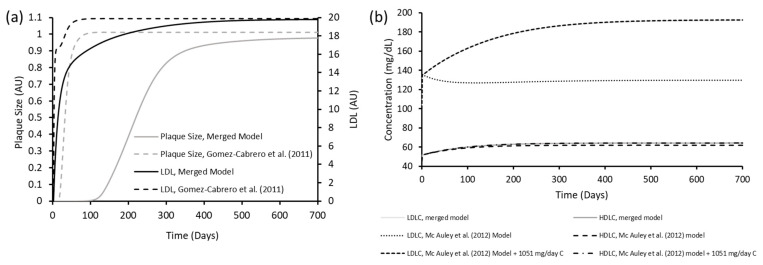
Comparison of results from the merged model with (**a**) Gomez-Cabrero et al. (2011) [72] and (**b**) Mc Auley et al. (2012) [56]. In (**a**), the grey solid line (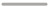) represents plaque size in the merged model and the grey dashed line (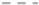) characterises plaque size in the Gomez-Cabrero et al. (2011) [72] model (left y-axis). The black solid line (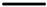) represents LDL in the merged model and the black dashed line (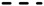) signifies LDL in the Gomez-Cabrero et al. (2011) [72] model (right y-axis). In (**b**), the light grey solid line (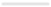) is representative of LDLC in the merged model, the round dot line (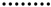) represents LDLC in the Mc Auley et al. (2012) [56] model, and the square dot line (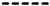) signifies LDLC in the Mc Auley et al. (2012) [56] model when DC was adjusted to 1051 mg/day. The dark grey line (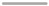) describes HDLC in the merged model, the dashed line (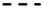) represents HDLC in the Mc Auley et al. (2012) [56] model, and the dash dot line (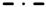) is representative of HDLC in the Mc Auley et al. (2012) [56] model when 1051 mg/day of cholesterol ingestion was simulated.

When the inferred parameters were included in the merged system, its output was similar to that of the parent models (Figure 4a,b). Figure 4a illustrates how the merged model compares with the Gomez-Cabrero et al. (2011) [72] model. In this model, LDL is reflective of the concentration within the intima. LDL in the merged model reaches a similar level to the Gomez-Cabrero et al. (2011) [72] model. Likewise, plaque size is comparable at 700 days, although this is slower to form in the merged model. A plaque value of zero is defined as the initial stage of plaque growth, and a value of one is defined as its final stage. Specifically, growth of the plaque starts with a lag period of no, or limited, growth until day ~100. After day ~100, growth increases exponentially before slowing at approximately day 400, where growth heads towards a steady state value of one.

Figure 4b compares the merged model with the Mc Auley et al. (2012) [56] model. LDL-C is recorded as mg/dL in line with the parent model, and refers to its concentration in the plasma. The initial concentration of LDL-C is 100 mg/dL in each case. In each of the three models, LDL-C rises to 136 mg/dL on day 1. However, in the Mc Auley et al. (2012) [56] model, LDL-C then begins to decline before reaching a steady state after approximately 100 days. Conversely, LDL-C increases within the merged model before reaching a steady state. After 700 days, LDL-C in the Mc Auley et al. (2012) [56] model is significantly lower than the merged model (130 vs. 192 mg/dL). However, once DC is increased to 1051 from 304 mg/day in the Mc Auley et al. (2012) [56] model, LDLC is comparable (193 mg/dL). HDL-C is similar between the merged model, and the Mc Auley (2012) [56] model with and without adjusted DC. In each case, HDL-C increases from 45 mg/dL to 60–62 mg/DL at day 150. HDL stays relatively level after this point reaching values of 62–64 mg/dL at day 700. 

### 3.2. Metabolic Control Analysis

A metabolic control analysis (MCA) was conducted in COPASI. Supplementary File S4 details the results of the MCA. Specifically, this file contains the flux control coefficient analysis and metabolic control coefficient analysis. As cholesterol absorption, excretion, hepatic synthesis are targets of pharmacological interventions we explored how perturbations to these reactions impacted the model variables [LDLC] and [PLAQUE] (Table 1). The concentration control coefficients in Table 1 demonstrate the impact of perturbations to the rate of hepatic cholesterol synthesis, cholesterol absorption, and cholesterol excretion has on the species [LDLC] and [PLAQUE]. In particular, [PLAQUE] is highly responsive to these perturbations. The positive scaled concentration control coefficients for hepatic cholesterol synthesis and cholesterol absorption indicate that as the rate of these reactions increase, so do [LDLC] and [PLAQUE]. Conversely negative scaled concentration control coefficients observed with cholesterol excretion demonstrate that as the rate of cholesterol excretion increases, [LDLC] and [PLAQUE] decrease.

### 3.3. Model Predictability: Therapeutic Interventions

It has been postulated that early and mid-stage atherosclerosis could be reversed via lowering dietary cholesterol or using a nutritional or pharmacological intervention [79]. The merged model was used to explore this possibility. The two interventions that were focused on are statins and plant sterols. Statins have previously been explored using the Mc Auley et al. (2012) model [70]. To do this, hepatic cholesterol synthesis was reduced by 75%. This reduction of 75% was calculated based on an oral dose of 40 mg/day of simvastatin [70]. This intervention was simulated by reducing the rate constant HCSmax in the reaction hepatic cholesterol synthesis from 500 to 125.

**Figure 5:**
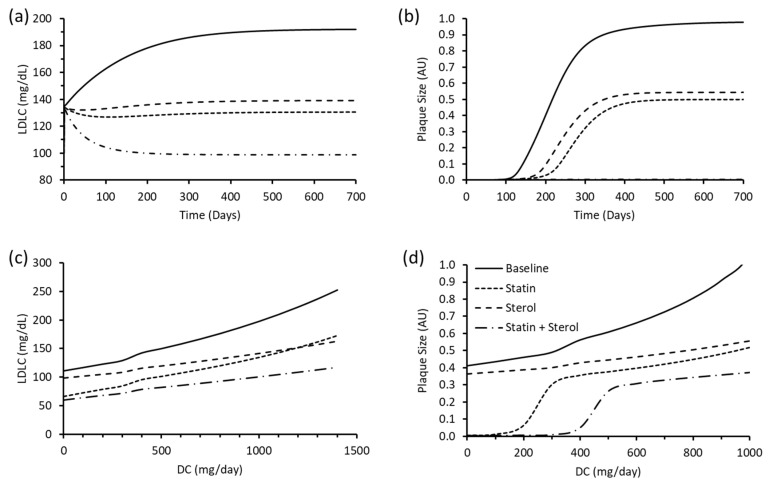
Impact of statin, sterol and combination therapy on (**a**) LDLC and (**b**) plaque size, and the impact of statin, sterol and combination therapy, dependent on DC, on (**c**) LDLC and (**d**) plaque size. Solid lines (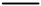) represent baseline values; square dot lines (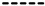) signify statin treatment; dashed lines (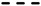) represent sterol treatment; and long dash dot lines (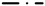) characterise combination therapy.

Plant sterols have been shown to reduce cholesterol absorption by 30–50% experimentally [80,81,82,83], with doses ranging from 50 µmol/L, 2% of dietary fat, and 1.5–2.2 mg/day. This intervention was simulated in the merged model by reducing the rate constant K6, from the cholesterol absorption reaction by 40% from 0.0005286 to 0.00031716. These simulations were run independently and in combination. The change in plaque size and LDLC was monitored over 700 days. Combined therapy had the greatest impact on LDLC and plaque size. Statin therapy was more effective at lowering LDLC and slowing the rate of plaque formation when compared to sterol therapy (Figure 5a,b).

Following this, the impact of these therapeutic interventions in the presence of different dietary regimes were assessed. Specifically, the above therapeutic strategies were simulated in the presence of DC ranging from 0 to 1500 mg/day for 700 days. Interval sizes of 100 mg/day were recorded. LDLC rose as the amount of DC increased and levels were greatest when no treatment was simulated. Sterols resulted in greater LDLC when compared to statins up to a DC value of approximately 1200 mg/day, where further DC resulted in sterols giving lower LDLC (Figure 5c). Plaque growth was greatest when no treatment strategy was simulated. Statins were more effective than sterols at slowing the rate of plaque growth up to DC values of 1200 mg/day. Plaque growth could be halted with statin therapy when DC was less than 100, and with combination therapy when DC was less than 300 mg/day (Figure 5d).

## 4. Discussion

Computational models are increasingly used to study complex biological systems. Many models exist which have captured various aspects of both cholesterol metabolism and atherosclerosis [53]. However, they do not fully capture these processes. The aim of this investigation was to overcome this gap in the field by combining a model of whole-body cholesterol metabolism and atherosclerosis. The merged model is an attempt to overcome the longstanding obstacle of combining two models which exist on different scales. This model was deposited in BioModels [65] and assigned the identifier MODEL2306300001. Here it will be available for future analysis and modification.

The merged model is capable of replicating behaviour consistent with the parent models. MCA of the merged model also revealed how changes to hepatic cholesterol synthesis, cholesterol absorption, and cholesterol excretion can affect plaque growth by raising circulating levels of LDL-C which ultimately drives atherosclerosis. The model was also able to recapitulate the findings of a previous study which utilised the Mc Auley et al. (2012) [56] model, to illustrate how statins affect LDL-C levels in addition to plaque growth [70]. It has been demonstrated in vivo that statins resulted in a 38% reduction in LDL-C after 6 weeks in hypercholesterolaemia patients. This change was maintained over a 1-year period [84]. Although the merged model showed a 13.3% reduction after 6 weeks, this rose to 31.2% after 1 year (Figure 5a). These results are akin to the results found by Palvaast et al. (2015) [70] after simulating statin treatment in silico. Specifically, it was found that a 75% reduction in hepatic cholesterol synthesis resulted in a 14% reduction in LDL-C after 6 weeks, which rose to 33% after 1 year. Likewise, the model showed that after 4 weeks, sterol treatment reduced LDL-C by 8.3%. This result is comparable to the results found from a double-blinded, randomised study of 59 hypercholesterolaemia subjects. In this study, it was demonstrated that ingestion of rapeseed sterol margarine for 4 weeks induced an 8.2% reduction in LDL-C [85]. Similarly, a meta-analysis has revealed that hypercholesterolaemia patients who took sterols concurrently (1.8–6 mg/day) with statins (40–80 mg/day) exhibited on average 13.26 mg/dL lower LDL-C (95% CI 9.18–17.34 mg/dL) compared to subjects on statins alone [86]. Of the 8 included studies, follow ups took place 4–16 weeks after treatment. When simulating combination therapy in the merged model, a 13.2–14.6 mg/dL reduction in LDL-C was observed after 6 and 7 weeks respectively. Between weeks 4 and 16, the merged model found that LDL-C was between 11.1–23.7 mg/dL lower than statin treatment alone (Table S4, Supplementary File S1).

The model also demonstrated the important role of dietary cholesterol in tandem with pharmaceutical interventions. The results from the merged model were similar to experimental data. For example, it was demonstrated that 4 weeks of a very-low-saturated-fat dairy and whole-wheat cereal diet, diet + statin, and the portfolio diet (containing plant sterols and viscous fibres) resulted in LDL-C levels of 159.7, 112.53, and 122.58 mg/dL respectively (9.6, 35.2, and 29.6% reductions in LDL-C after 4 weeks). These diets contained 28, 33, and 55 mg/day of cholesterol respectively [87]. When this was simulated in the merged model, after 4 weeks, LDL-C reduced to 125.69, 111.92 and 119.77 mg/dL respectively, following a similar trend to the experimental data (Figure S3, Supplementary File S1). 

Despite these findings, the combined model has several limitations which are worth outlining. The merged model was developed from two SBML encoded models. However, many other models exist which are not encoded in this framework. These models potentially have mechanisms which would add value to the merged model. For instance, the merged model does not include the dynamics of blood flow and the anatomical features of the artery. Atherosclerosis tends to occur at bifurcations within medium sized arteries [88]. Thus, the merged model does not represent these key physiological and anatomical features which are important to atherogenesis. Nor does the merged model completely represent the immuno-pathophysiology of atherosclerosis [89]. A further limitation is that the model has been simulated over a relatively short period of 700 days, whereas atherosclerosis is a long-term disease that can span many decades, with advancing age the major risk factor [90]. These disadvantages should be looked upon as a challenge to the community to continue the development of this area.

In spite of the disadvantages of this model and the challenges which persist in the field more broadly, there is a real need to continue developing models. A significant reason for using computational models is the growing need to reduce the number of animals used in research. Animal models are routinely employed to investigate both cholesterol metabolism and ACVD [91,92]. However, there is an ethical imperative to not use animals [93]. Furthermore, it is important to recognise that animals are imperfect analogues for humans [94]. Therefore, it is our hope that modelling will be used to a much greater extent in this field to overcome these issues.

## 5. Conclusions

This novel system combines whole-body cholesterol metabolism with atherosclerosis, and is capable of performing in silico experimentations. The merged model is effective at investigating therapeutic avenues to lower LDL-C and retard atherogenesis. It is hoped this merged model will act as a meaningful template for future work, which aims to further examine the nexus between cholesterol metabolism and atherosclerosis.

## Figures and Tables

**Table 1:** Metabolic control coefficient analysis of 3 pharmacological targets.

Species	Hepatic Cholesterol Synthesis	Cholesterol Absorption	Cholesterol Excretion
LDLC	0.0471523	0.786053	−0.760712
Plaque	1.28131	2.136	−2.06714

## Data Availability

The data presented in this study are available in the supplementary files. The merged model SBML is available on the BioModels database.

## Supplementary Materials

The following supporting information can be downloaded at: https://www.mdpi.com/article/10.3390/biology12081133/s1, Supplementary File S1: Supplementary Figures & Tables. Figure S1: SBGN diagram of the Mc Auley et al. (2012) [56] whole-body cholesterol model; Figure S2: BioPAX diagram of the Gomez-Cabrero et al. (2011) [72] model after importation into Vanted; Figure S3: Impact of dietary cholesterol in tandem with pharmaceutical interventions; Table S1: Converted global quantity values from week^−1^ to day^−1^; Table S2: Model species; Table S3: Model reactions; Table S4: Impact of statin, sterol, and combination therapy on LDL-C. Supplementary File S2: Model ODEs; Supplementary File S3: Merged Model COPASI file; Supplementary File S4: MCA.
