# Supplementary material for: Computationally Modelling Cholesterol Metabolism and Atherosclerosis
Source: Biology (Basel). 2023 Aug 14;12(8):1133. doi: 10.3390/biology12081133 (PMC10452179; doi:10.3390/biology12081133)
Supplement: Supplementary file 1 [file biology-12-01133-s001.zip › Supplementary file 1.pdf]

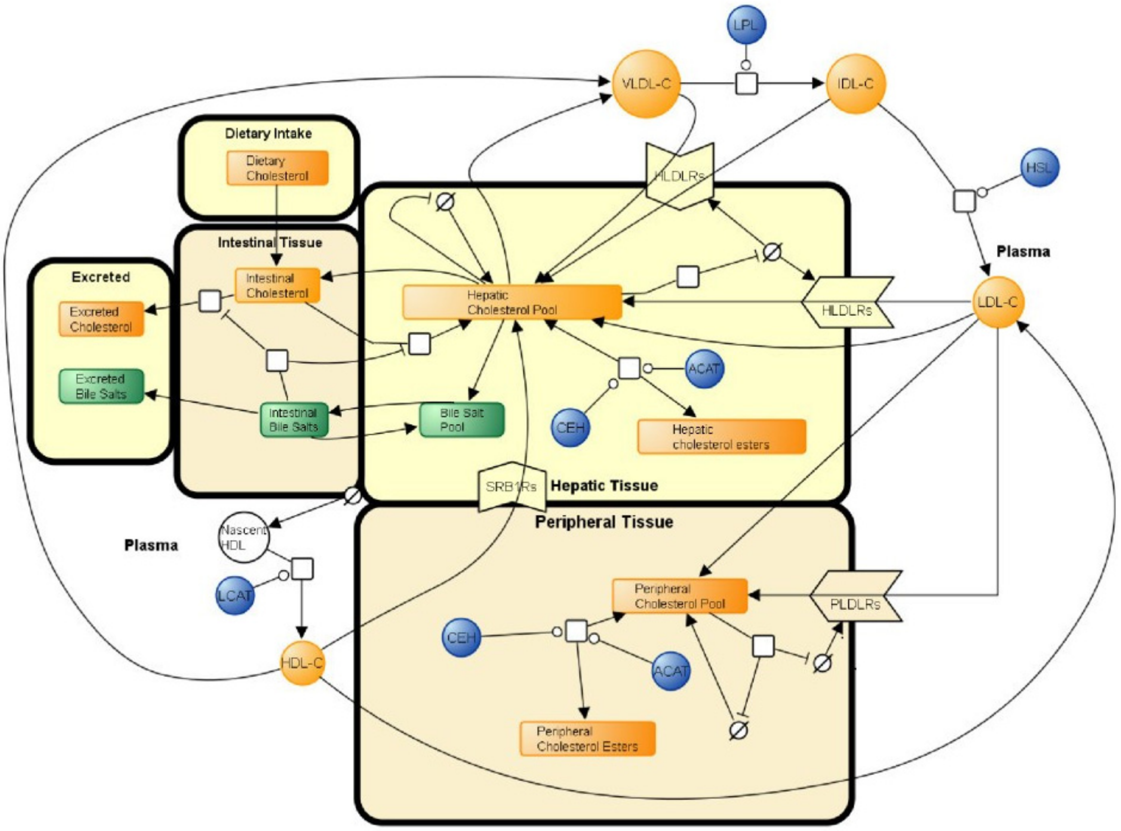

Figure S1. SBGN diagram of the Mc Auley et al. (2012) [56] whole-body cholesterol model.

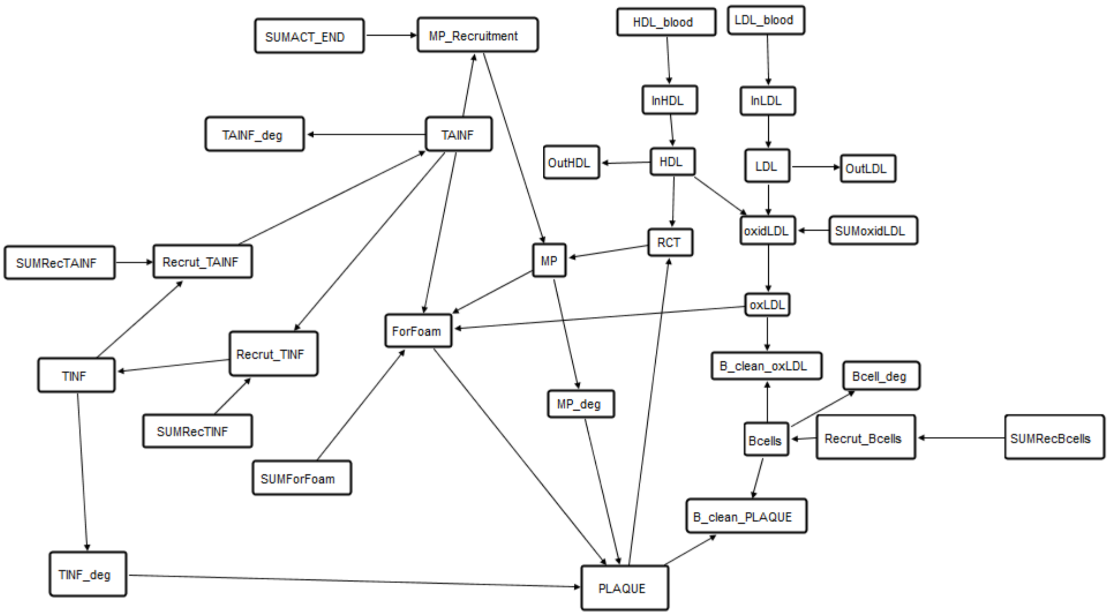

Figure S2. BioPAX diagram of the Gomez-Cabrero et al. (2011) [72] model after importation into Vanted.

Table S1. Converted global quantity values from week^{-1} to day^{-1}.

Global Quantity	Week^{-1} Value	Day^{-1} Value
ActEndConstant	0.147264	0.021038
ForFoamConstant	0.571007	0.081572
KBcleanoxLDL	0.150145	0.021449
KBcleanPLAQUE	0.431588	0.061655
Kdeg_Bcells	0.555978	0.079425
Kdeg_MP	0.196692	0.028099
Kdeg_TAINF	0.191241	0.027320
Kdeg_TINF	0.0882042	0.012601
KinHDL	1.0614	0.151629
KinLDL	4.06157	0.580224
KoutHDL	0.375371	0.053624
KoutLDL	0.55296	0.078994
KRCT	0.302073	0.043153
oxLDLConstant	0.28495	0.040707
RecBcellsConstant	1.14614	0.163734
RecTAINFConstant	3.20264	0.457520
RecTINFConstant	2.04297	0.291853

Table S2. Model species.

#	Name	Compartment	Type	Unit	Initial Concentration [Unit]
1	DC	Intake	fixed	1	1051
2	IC	Intestine	reactions	1	3150
3	ICS	Intestine	fixed	1	0
4	HBS	HepaticTissue	reactions	1	400
5	IBS	Intestine	reactions	1	467
6	EBS	Excreted	fixed	1	0
7	HFC	HepaticTissue	reactions	1	60000
8	EC	Excreted	fixed	1	0
9	INHDLS	Plasma	fixed	1	0
10	NHDL	Plasma	reactions	1	100
11	PFC	PeripheralTissue	reactions	1	57516
12	HCS	HepaticTissue	fixed	1	0
13	HCE	HepaticTissue	reactions	1	10000
14	ACAT	HepaticTissue	reactions	1	100
15	CEH	HepaticTissue	reactions	1	100
16	HNHDLS	HepaticTissue	fixed	1	0
17	VLDLC	Plasma	reactions	1	20
18	HLDLRs	HepaticTissue	reactions	1	100
19	HLDLRsS	HepaticTissue	fixed	1	600
20	HLDLRD	HepaticTissue	fixed	1	0
21	IDLC	Plasma	reactions	1	20
22	LPL	Plasma	reactions	1	100
23	LDLC	Plasma	reactions	1	100
24	HSL	Plasma	reactions	1	100
25	PLDLRs	PeripheralTissue	reactions	1	100
26	PLDLRsS	PeripheralTissue	fixed	1	575.16
27	PLDLRD	PeripheralTissue	fixed	1	0
28	PCE	PeripheralTissue	reactions	1	9363
29	PSS	PeripheralTissue	fixed	1	0
30	HDLC	Plasma	reactions	1	45
31	LCAT	Plasma	reactions	1	100
32	PCS	PeripheralTissue	fixed	1	0
33	CETP	Plasma	reactions	1	100

34	SRB1	HepaticTissue	reactions	1	100
35	LDL	Endothelium	reactions	1	0
36	oxLDL	Endothelium	reactions	1	0
37	TINF	Endothelium	reactions	1	0
38	SUMRecTAINF	Endothelium	assignment	1	0
39	TAINF	Endothelium	reactions	1	0
40	MP	Endothelium	reactions	1	0
41	SIGMOID_ACTENDstim	Endothelium	assignment	1	7.69165e-07
42	SUMACT_END	Endothelium	assignment	1	0
43	PLAQUE	Endothelium	reactions	1	0
44	SIGMOID_ACTENDinh	Endothelium	assignment	1	0.000423556
45	Bcells	Endothelium	reactions	1	0
46	SUMRecBcells	Endothelium	assignment	1	3.82182e-08
47	ACT_END	Endothelium	assignment	1	1.61748e-08
48	SUMoxidLDL	Endothelium	assignment	1	2.51464e-08
49	SUMForFoam	Endothelium	assignment	1	1.09875e-08
50	SUMRecTINF	Endothelium	assignment	1	2.96687e-08
51	HDL	Endothelium	reactions	1	0

Table S3. Model reactions.

#	Name	Reaction
1	Ingestion	DC -> IC; DC
2	Intestinal Cholesterol Synthesis	ICS -> IC; IC
3	Bile Salt Release	HBS -> IBS; HBS
4	Bile Salt Return	IBS -> HBS; IBS
5	Bile Salt Excretion	IBS -> EBS; IBS
6	Bile Salt Synthesis	HFC -> HBS; HBS HFC
7	Cholesterol Absorption	IC -> HFC; IBS IC
8	Cholesterol Excretion	IC -> EC; IBS IC
9	Intestinal Nascent HDL Synthesis	INHDLS -> NHDL; PFC
10	Billary Cholesterol Release	HFC -> IC; HFC
11	Hepatic Cholesterol Synthesis	HCS -> HFC; HFC
12	Hepatic Cholesterol Storage	HFC -> HCE; ACAT HFC
13	Release of Stored Cholesterol	HCE -> HFC; CEH HCE
14	Hepatic Nascent HDL Synthesis	HNHDLS -> NHDL; PFC
15	VLDL Cholesterol Formation	HFC -> VLDLC; HFC
16	Hepatic LDLR Synthesis	HLDLRsS -> HLDLRs; HLDLRsS HFC
17	Hepatic LDL Receptor Degradation	HLDLRs -> HLDLRD; HLDLRs
18	VLDL Cholesterol ReUptake	VLDLC -> HFC; VLDLC
19	IDL Cholesterol Formation	VLDLC -> IDLC; VLDLC LPL
20	IDL Cholesterol ReUptake	IDLC -> HFC; IDLC
21	LDL Cholesterol Formation	IDLC -> LDLC; HSL IDLC
22	Receptor Dependent Hepatic Uptake	LDLC -> HFC; HLDLRs LDLC
23	Receptor Independent Hepatic Uptake	LDLC -> HFC; LDLC
24	Receptor Dependent Peripheral Uptake	LDLC -> PFC; PLDLRs LDLC
25	Receptor Independent Peripheral Uptake	LDLC -> PFC; LDLC
26	Peripheral LDLR Synthesis	PLDLRsS -> PLDLRs; PFC PLDLRsS
27	Peripheral LDL Receptor Degradation	PLDLRs -> PLDLRD; PLDLRs
28	Peripheral Cholesterol Storage	PFC -> PCE; ACAT PFC
29	Release of Stored Peripheral Cholesterol	PCE -> PFC; CEH PCE
30	Peripheral Steroid Production	PFC -> PSS; PFC
31	HDL Cholesterol Formation	PFC + NHDL -> HDLC; LCAT PFC NHDL
32	Peripheral Cholesterol Synthesis	PCS -> PFC; PFC
33	CETP Mediated Transfer To VLDL	HDLC -> VLDLC; CETP HDLC

34	CETP Mediated TransferTo LDL	HDLC -> LDLC; CETP HDLC
35	Reverse Cholesterol Transport	HDLC -> HFC; SRB1 HDLC
36	Bcell_deg	Bcells ->
37	InLDL	LDLC -> LDL
38	OutLDL	LDL ->
39	MP_deg	MP -> PLAQUE
40	OutHDL	HDL ->
41	TAINF_deg	TAINF ->
42	oxidLDL	LDL -> oxLDL; SUMoxidLDL HDL
43	TINF_deg	TINF -> PLAQUE
44	InHDL	HDLC -> HDL
45	B_clean_oxLDL	Bcells + oxLDL ->
46	B_clean_PLAQUE	Bcells + PLAQUE ->
47	RCT[merge]	HDL + PLAQUE -> MP
48	Recrut_TINF	-> TINF; SUMRecTINF TAINF
49	Recrut_TAINF	-> TAINF; SUMRecTAINF TINF
50	Recrut_Bcells	-> Bcells; SUMRecBcells
51	MP_Recruitment	-> MP; SUMACT_END TAINF
52	ForFoam[merge]	MP + oxLDL -> PLAQUE; SUMForFoam TAINF

Table S4. Impact of statin, sterol, and combination therapy on LDL-C.

Time		LDL-C (mg/dL)			
Day	Week	Baseline	Statin	Sterol	Combination
0	0	100.000	100.000	100.000	100.000
7	1	136.970	133.008	133.331	129.405
14	2	139.514	131.846	132.816	125.332
21	3	141.949	130.889	132.520	121.907
28	4	144.256	130.081	132.329	118.963
35	5	146.449	129.403	132.220	116.427
42	6	148.540	128.840	132.178	114.241
49	7	150.537	128.376	132.192	112.351
56	8	152.449	127.999	132.252	110.715
63	9	154.281	127.697	132.349	109.296
70	10	156.038	127.461	132.476	108.062
77	11	157.725	127.282	132.626	106.986
84	12	159.344	127.151	132.795	106.046
91	13	160.899	127.062	132.979	105.223
98	14	162.392	127.008	133.173	104.501
105	15	163.825	126.986	133.376	103.867
112	16	165.200	126.989	133.583	103.307
119	17	166.520	127.014	133.793	102.813
126	18	167.786	127.058	134.005	102.377
133	19	169.000	127.116	134.216	101.990
140	20	170.163	127.188	134.426	101.647
147	21	171.276	127.269	134.633	101.341
154	22	172.342	127.359	134.837	101.070
161	23	173.362	127.455	135.036	100.828
168	24	174.337	127.557	135.231	100.612
175	25	175.269	127.661	135.421	100.419
182	26	176.159	127.769	135.605	100.246
189	27	177.008	127.877	135.783	100.091
196	28	177.818	127.987	135.955	99.953
203	29	178.591	128.096	136.121	99.828
210	30	179.326	128.204	136.282	99.717
217	31	180.027	128.311	136.435	99.616
224	32	180.694	128.417	136.583	99.526
231	33	181.328	128.520	136.725	99.445
238	34	181.931	128.621	136.860	99.372
245	35	182.504	128.719	136.990	99.307
252	36	183.049	128.815	137.114	99.247
259	37	183.565	128.908	137.232	99.194
266	38	184.055	128.997	137.345	99.146
273	39	184.520	129.084	137.452	99.103
280	40	184.961	129.167	137.554	99.063
287	41	185.378	129.247	137.651	99.028

294	42	185.773	129.324	137.743	98.996
301	43	186.148	129.398	137.831	98.967
308	44	186.502	129.469	137.914	98.941
315	45	186.837	129.536	137.992	98.917
322	46	187.154	129.601	138.067	98.896
329	47	187.453	129.663	138.137	98.877
336	48	187.736	129.721	138.204	98.859
343	49	188.003	129.777	138.267	98.844
350	50	188.255	129.830	138.326	98.829
357	51	188.493	129.881	138.382	98.816
364	52	188.718	129.929	138.435	98.804
365	52	188.749	129.935	138.443	98.803

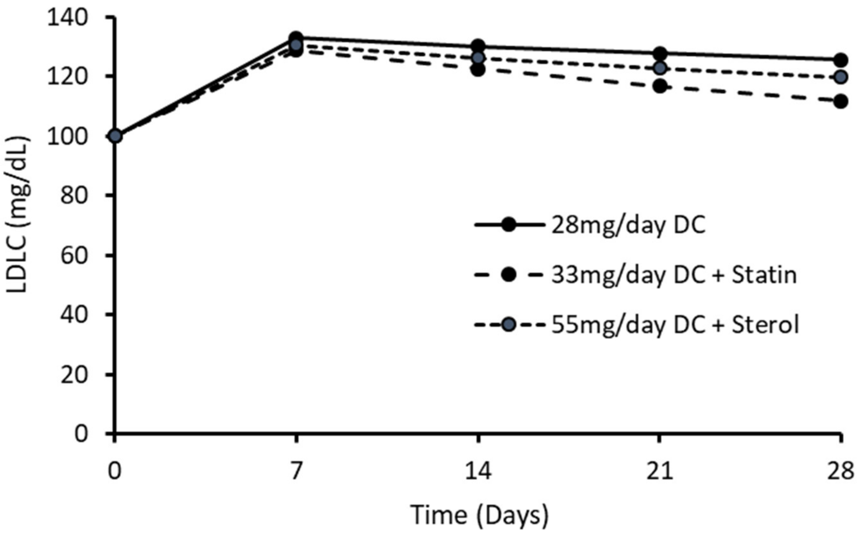

Figure S3. Impact of dietary cholesterol in tandem with pharmaceutical interventions.
