# Supplementary material for: Computationally Modelling Cholesterol Metabolism and Atherosclerosis
Source: Biology (Basel). 2023 Aug 14;12(8):1133. doi: 10.3390/biology12081133 (PMC10452179; doi:10.3390/biology12081133)
Supplement: Supplementary file 1 [file biology-12-01133-s001.zip › Supplmentary file 2 ODEs.pdf]

$$\frac{d([\mathrm{IC}]\cdot V_{\mathrm{Intestine}})}{dt} = +(1\cdot[\mathrm{DC}]) + V_{\mathrm{Intestine}}\cdot\left(\frac{\text{""Rate Law for Intestinal Cholesterol Synthesis""}(100, [\mathrm{IC}], 3120, 5)}{V_{\mathrm{Intestine}}}\right) - (0.0005286\cdot[\mathrm{IC}]\cdot[\mathrm{IBS}]) - (0.0005286\cdot[\mathrm{IC}]\cdot[\mathrm{IBS}]) + \left(\frac{2000}{1+\left(\frac{55326}{[\mathrm{HFC}]}\right)^{5}}\right)$$

$$\frac{d([\mathrm{HBS}]\cdot V_{\mathrm{HepaticTissue}})}{dt} = -(6\cdot[\mathrm{HBS}]) + (4.29\cdot[\mathrm{IBS}]) + V_{\mathrm{HepaticTissue}}\cdot\left(\frac{\text{""Rate Law for Bile Salt Synthesis""}(2.66, [\mathrm{HFC}], [\mathrm{HBS}])}{V_{\mathrm{HepaticTissue}}}\right)$$

$$\frac{d([\mathrm{IBS}]\cdot V_{\mathrm{Intestine}})}{dt} = +(6\cdot[\mathrm{HBS}]) - (4.29\cdot[\mathrm{IBS}]) - (0.856\cdot[\mathrm{IBS}])$$

$$\frac{d([\mathrm{HFC}]\cdot V_{\mathrm{HepaticTissue}})}{dt} = +V_{\mathrm{HepaticTissue}}\cdot\left(\frac{\text{""Rate Law for Hepatic Cholesterol Synthesis""}(500, [\mathrm{HFC}], 93925, 5)}{V_{\mathrm{HepaticTissue}}}\right) - V_{\mathrm{HepaticTissue}}\cdot\left(\frac{\text{""Rate Law for Hepatic Cholesterol Storage\_1""}(1, [\mathrm{ACAT}], [\mathrm{HFC}])}{V_{\mathrm{HepaticTissue}}}\right) + V_{\mathrm{HepaticTissue}}\cdot\left(\frac{\text{""Rate Law for Release of Stored Cholesterol""}(5.998, [\mathrm{CEH}], [\mathrm{HCE}])}{V_{\mathrm{HepaticTissue}}}\right) - (0.016\cdot[\mathrm{HFC}]) + (0.0496\cdot[\mathrm{VLDLC}]) + (0.054\cdot[\mathrm{IDLC}]) + (0.068\cdot[\mathrm{LDLC}]\cdot[\mathrm{HLDLRs}]) + (0.005\cdot[\mathrm{LDLC}]) + (0.05\cdot[\mathrm{HDLC}]\cdot[\mathrm{SRB1}]) - V_{\mathrm{HepaticTissue}}\cdot\left(\frac{\text{""Rate Law for Bile Salt Synthesis""}(2.66, [\mathrm{HFC}], [\mathrm{HBS}])}{V_{\mathrm{HepaticTissue}}}\right) + (0.0005286\cdot[\mathrm{IC}]\cdot[\mathrm{IBS}]) - \left(\frac{2000}{1+\left(\frac{55326}{[\mathrm{HFC}]}\right)^{5}}\right)$$

$$\frac{d([\mathrm{NHDL}]\cdot V_{\mathrm{Plasma}})}{dt} = +(0.005\cdot[\mathrm{PFC}]) - (1.5\mathrm{e}{-05}\cdot[\mathrm{PFC}]\cdot[\mathrm{NHDL}]\cdot[\mathrm{LCAT}]) + (0.0005\cdot[\mathrm{PFC}])$$

$$\frac{d([\mathrm{PFC}]\cdot V_{\mathrm{PeripheralTissue}})}{dt} = +(0.00675\cdot[\mathrm{PLDLRs}]\cdot[\mathrm{LDLC}]) + (5\mathrm{e}{-06}\cdot[\mathrm{LDLC}]) - (0.017386\cdot[\mathrm{ACAT}]\cdot[\mathrm{PFC}]) + (0.1068\cdot[\mathrm{CEH}]\cdot[\mathrm{PCE}]) - V_{\mathrm{PeripheralTissue}}\cdot\left(\frac{0.0005\cdot[\mathrm{PFC}]}{V_{\mathrm{PeripheralTissue}}}\right) - (1.5\mathrm{e}{-05}\cdot[\mathrm{PFC}]\cdot[\mathrm{NHDL}]\cdot[\mathrm{LCAT}]) + V_{\mathrm{PeripheralTissue}}\cdot\left(\frac{\text{""Rate Law for Peripheral Cholesterol Synthesis""}(500, [\mathrm{PFC}], 80342, 5)}{V_{\mathrm{PeripheralTissue}}}\right)$$

$$\frac{d([\mathrm{HCE}]\cdot V_{\mathrm{HepaticTissue}})}{dt} = +V_{\mathrm{HepaticTissue}}\cdot\left(\frac{\text{""Rate Law for Hepatic Cholesterol Storage\_1""}(1, [\mathrm{ACAT}], [\mathrm{HFC}])}{V_{\mathrm{HepaticTissue}}}\right) - V_{\mathrm{HepaticTissue}}\cdot\left(\frac{\text{""Rate Law for Release of Stored Cholesterol""}(5.998, [\mathrm{CEH}], [\mathrm{HCE}])}{V_{\mathrm{HepaticTissue}}}\right)$$

$$\frac{d([\mathrm{VLDLC}]\cdot V_{\mathrm{Plasma}})}{dt} = +(0.016\cdot[\mathrm{HFC}]) - (0.0496\cdot[\mathrm{VLDLC}]) - V_{\mathrm{Plasma}}\cdot\left(\frac{\text{""Rate Law for IDL Cholesterol Formation""}(0.43, [\mathrm{VLDLC}], [\mathrm{LPL}])}{V_{\mathrm{Plasma}}}\right) + V_{\mathrm{Plasma}}\cdot\left(\frac{\text{""Rate Law for CETP Mediated Transfer To VLDL""}(0.01, [\mathrm{HDLC}], [\mathrm{CETP}])}{V_{\mathrm{Plasma}}}\right)$$

$$\frac{d([\mathrm{HLDLRs}]\cdot V_{\mathrm{HepaticTissue}})}{dt} = +V_{\mathrm{HepaticTissue}}\cdot\left(\frac{\text{""Rate Law for Hepatic LDLR Synthesis""}(100, [\mathrm{HLDLRsS}], [\mathrm{HFC}])}{V_{\mathrm{HepaticTissue}}}\right) - V_{\mathrm{HepaticTissue}}\cdot\left(\frac{0.01\cdot[\mathrm{HLDLRs}]}{V_{\mathrm{HepaticTissue}}}\right)$$

$$\frac{d([\mathrm{IDLC}]\cdot V_{\mathrm{Plasma}})}{dt} = +V_{\mathrm{Plasma}}\cdot\left(\frac{\text{""Rate Law for IDL Cholesterol Formation""}(0.43, [\mathrm{VLDLC}], [\mathrm{LPL}])}{V_{\mathrm{Plasma}}}\right) - (0.054\cdot[\mathrm{IDLC}]) - V_{\mathrm{Plasma}}\cdot\left(\frac{\text{""Rate Law for LDL Cholesterol Formation""}(0.38, [\mathrm{IDLC}], [\mathrm{HSL}])}{V_{\mathrm{Plasma}}}\right)$$

$$\frac{d([\mathrm{LDLC}]\cdot V_{\mathrm{Plasma}})}{dt} = +V_{\mathrm{Plasma}}\cdot\left(\frac{\text{""Rate Law for LDL Cholesterol Formation""}(0.38, [\mathrm{IDLC}], [\mathrm{HSL}])}{V_{\mathrm{Plasma}}}\right) - (0.068\cdot[\mathrm{LDLC}]\cdot[\mathrm{HLDLRs}]) - (0.005\cdot[\mathrm{LDLC}]) - (0.00675\cdot[\mathrm{PLDLRs}]\cdot[\mathrm{LDLC}]) - (5\mathrm{e}{-06}\cdot[\mathrm{LDLC}]) + V_{\mathrm{Plasma}}\cdot\left(\frac{\text{""Rate Law for CETP Mediated TransferTo LDL""}(0.001, [\mathrm{HDLC}], [\mathrm{CETP}])}{V_{\mathrm{Plasma}}}\right) - V_{\mathrm{Endothelium}}\cdot(\mathrm{KinLDL}\cdot[\mathrm{LDLC}]\cdot M)$$

$$\frac{d([\mathrm{PLDLRs}]\cdot V_{\mathrm{PeripheralTissue}})}{dt} = +V_{\mathrm{PeripheralTissue}}\cdot\left(\frac{\text{""Rate Law for Peripheral LDLR Synthesis""}(100, [\mathrm{PLDLRsS}], [\mathrm{PFC}])}{V_{\mathrm{PeripheralTissue}}}\right) - V_{\mathrm{PeripheralTissue}}\cdot\left(\frac{0.01\cdot[\mathrm{PLDLRs}]}{V_{\mathrm{PeripheralTissue}}}\right)$$

$$\frac{d([\mathrm{PCE}]\cdot V_{\mathrm{PeripheralTissue}})}{dt} = +(0.017386\cdot[\mathrm{ACAT}]\cdot[\mathrm{PFC}]) - (0.1068\cdot[\mathrm{CEH}]\cdot[\mathrm{PCE}])$$

$$\frac{d([\mathrm{HDLC}]\cdot V_{\mathrm{Plasma}})}{dt} = +(1.5\mathrm{e}{-05}\cdot[\mathrm{PFC}]\cdot[\mathrm{NHDL}]\cdot[\mathrm{LCAT}]) - V_{\mathrm{Plasma}}\cdot\left(\frac{\text{""Rate Law for CETP Mediated Transfer To VLDL""}(0.01, [\mathrm{HDLC}], [\mathrm{CETP}])}{V_{\mathrm{Plasma}}}\right) - V_{\mathrm{Plasma}}\cdot\left(\frac{\text{""Rate Law for CETP Mediated TransferTo LDL""}(0.001, [\mathrm{HDLC}], [\mathrm{CETP}])}{V_{\mathrm{Plasma}}}\right) - (0.05\cdot[\mathrm{HDLC}]\cdot[\mathrm{SRB1}]) - V_{\mathrm{Endothelium}}\cdot(\mathrm{KinHDL}\cdot[\mathrm{HDLC}]\cdot M)$$

$$\frac{d([\mathrm{LDL}]\cdot V_{\mathrm{Endothelium}})}{dt} = +V_{\mathrm{Endothelium}}\cdot(\mathrm{KinLDL}\cdot[\mathrm{LDLC}]\cdot M) - V_{\mathrm{Endothelium}}\cdot(\mathrm{KoutLDL}\cdot[\mathrm{LDL}]) - V_{\mathrm{Endothelium}}\cdot\left(\mathrm{oxLDLConstant}\cdot\left(\mathrm{oxLDLstim\_a}+\frac{1-\mathrm{oxLDLstim\_a}}{1+e^{\frac{\frac{-4\cdot \mathrm{oxLDLstim\_b}}{\mathrm{oxLDLstim\_c}}}{1-\mathrm{oxLDLstim\_a}}\cdot([\mathrm{SUMoxidLDL}]-\mathrm{oxLDLstim\_c})}}\right)\cdot\left(1-\mathrm{oxLDLinh\_a}-\frac{1-\mathrm{oxLDLinh\_a}}{1+e^{\frac{\frac{-4\cdot \mathrm{oxLDLinh\_b}}{\mathrm{oxLDLinh\_c}}}{1-\mathrm{oxLDLinh\_a}}\cdot([\mathrm{HDL}]-\mathrm{oxLDLinh\_c})}}\right)\cdot[\mathrm{LDL}]\right)$$

$$\frac{d([\mathrm{oxLDL}]\cdot V_{\mathrm{Endothelium}})}{dt} = +V_{\mathrm{Endothelium}}\cdot\left(\mathrm{oxLDLConstant}\cdot\left(\mathrm{oxLDLstim\_a}+\frac{1-\mathrm{oxLDLstim\_a}}{1+e^{\frac{\frac{-4\cdot \mathrm{oxLDLstim\_b}}{\mathrm{oxLDLstim\_c}}}{1-\mathrm{oxLDLstim\_a}}\cdot([\mathrm{SUMoxidLDL}]-\mathrm{oxLDLstim\_c})}}\right)\cdot\left(1-\mathrm{oxLDLinh\_a}-\frac{1-\mathrm{oxLDLinh\_a}}{1+e^{\frac{\frac{-4\cdot \mathrm{oxLDLinh\_b}}{\mathrm{oxLDLinh\_c}}}{1-\mathrm{oxLDLinh\_a}}\cdot([\mathrm{HDL}]-\mathrm{oxLDLinh\_c})}}\right)\cdot[\mathrm{LDL}]\right) - V_{\mathrm{Endothelium}}\cdot(\mathrm{KBcleanoxLDL}\cdot[\mathrm{Bcells}]\cdot[\mathrm{oxLDL}]) - V_{\mathrm{Endothelium}}\cdot\left(\mathrm{ForFoamConstant}\cdot\left(\mathrm{ForFoamstim\_a}+\frac{1-\mathrm{ForFoamstim\_a}}{1+e^{\frac{\frac{-4\cdot \mathrm{ForFoamstim\_b}}{\mathrm{ForFoamstim\_c}}}{1-\mathrm{ForFoamstim\_a}}\cdot([\mathrm{SUMForFoam}]-\mathrm{ForFoamstim\_c})}}\right)\cdot\left(1-\mathrm{ForFoaminh\_a}-\frac{1-\mathrm{ForFoaminh\_a}}{1+e^{\frac{\frac{-4\cdot \mathrm{ForFoamstim\_b}}{\mathrm{ForFoaminh\_c}}}{1-\mathrm{ForFoaminh\_a}}\cdot([\mathrm{TAINF}]-\mathrm{ForFoaminh\_c})}}\right)\cdot[\mathrm{MP}]\cdot[\mathrm{oxLDL}]\right)$$

$$\frac{d([\mathrm{TINF}]\cdot V_{\mathrm{Endothelium}})}{dt} = -V_{\mathrm{Endothelium}}\cdot(\mathrm{Kdeg\_TINF}\cdot[\mathrm{TINF}]) + V_{\mathrm{Endothelium}}\cdot\left(\mathrm{RecTINFConstant}\cdot\left(\mathrm{RecTINFstim\_a}+\frac{1-\mathrm{RecTINFstim\_a}}{1+e^{\frac{\frac{-4\cdot \mathrm{RecTINFstim\_b}}{\mathrm{RecTINFstim\_c}}}{1-\mathrm{RecTINFstim\_a}}\cdot([\mathrm{SUMRecTINF}]-\mathrm{RecTINFstim\_c})}}\right)\cdot\left(1-\mathrm{RecTINFinh\_a}-\frac{1-\mathrm{RecTINFinh\_a}}{1+e^{\frac{\frac{-4\cdot \mathrm{RecTINFinh\_b}}{\mathrm{RecTINFinh\_c}}}{1-\mathrm{RecTINFinh\_a}}\cdot([\mathrm{TAINF}]-\mathrm{RecTINFinh\_c})}}\right)\right)$$

$$[\mathrm{SUMRecTAINF}] = [\mathrm{TAINF}] + \mathrm{alfa7}\cdot[\mathrm{MP}]$$

$$\frac{d([\mathrm{TAINF}]\cdot V_{\mathrm{Endothelium}})}{dt} = -V_{\mathrm{Endothelium}}\cdot(\mathrm{Kdeg\_TAINF}\cdot[\mathrm{TAINF}]) + V_{\mathrm{Endothelium}}\cdot\left(\mathrm{RecTAINFConstant}\cdot\left(\mathrm{RecTAINFstim\_a}+\frac{1-\mathrm{RecTAINFstim\_a}}{1+e^{\frac{\frac{-4\cdot \mathrm{RecTAINFstim\_b}}{\mathrm{RecTAINFstim\_c}}}{1-\mathrm{RecTAINFstim\_a}}\cdot([\mathrm{SUMRecTAINF}]-\mathrm{RecTAINFstim\_c})}}\right)\cdot\left(1-\mathrm{RecTAINFinh\_a}-\frac{1-\mathrm{RecTAINFinh\_a}}{1+e^{\frac{\frac{-4\cdot \mathrm{RecTAINFinh\_b}}{\mathrm{RecTAINFinh\_c}}}{1-\mathrm{RecTAINFinh\_a}}\cdot([\mathrm{TINF}]-\mathrm{RecTAINFinh\_c})}}\right)\right)$$

$$\frac{d([\mathrm{MP}]\cdot V_{\mathrm{Endothelium}})}{dt} = -V_{\mathrm{Endothelium}}\cdot(\mathrm{Kdeg\_MP}\cdot[\mathrm{MP}]) + V_{\mathrm{Endothelium}}\cdot(\mathrm{KRCT}\cdot[\mathrm{HDL}]\cdot[\mathrm{PLAQUE}]) + V_{\mathrm{Endothelium}}\cdot\left(\mathrm{ActEndConstant}\cdot\left(\mathrm{ActEndstim\_a}+\frac{1-\mathrm{ActEndstim\_a}}{1+e^{\frac{\frac{-4\cdot \mathrm{ActEndstim\_b}}{\mathrm{ActEndstim\_c}}}{1-\mathrm{ActEndstim\_a}}\cdot([\mathrm{SUMACT\_END}]-\mathrm{ActEndstim\_c})}}\right)\cdot\left(1-\mathrm{ActEndinh\_a}-\frac{1-\mathrm{ActEndinh\_a}}{1+e^{\frac{\frac{-4\cdot \mathrm{ActEndinh\_b}}{\mathrm{ActEndinh\_c}}}{1-\mathrm{ActEndinh\_a}}\cdot([\mathrm{TAINF}]-\mathrm{ActEndinh\_c})}}\right)\right) - V_{\mathrm{Endothelium}}\cdot\left(\mathrm{ForFoamConstant}\cdot\left(\mathrm{ForFoamstim\_a}+\frac{1-\mathrm{ForFoamstim\_a}}{1+e^{\frac{\frac{-4\cdot \mathrm{ForFoamstim\_b}}{\mathrm{ForFoamstim\_c}}}{1-\mathrm{ForFoamstim\_a}}\cdot([\mathrm{SUMForFoam}]-\mathrm{ForFoamstim\_c})}}\right)\cdot\left(1-\mathrm{ForFoaminh\_a}-\frac{1-\mathrm{ForFoaminh\_a}}{1+e^{\frac{\frac{-4\cdot \mathrm{ForFoamstim\_b}}{\mathrm{ForFoaminh\_c}}}{1-\mathrm{ForFoaminh\_a}}\cdot([\mathrm{TAINF}]-\mathrm{ForFoaminh\_c})}}\right)\cdot[\mathrm{MP}]\cdot[\mathrm{oxLDL}]\right)$$

$$[\mathrm{SIGMOID\_ACTENDstim}] = \mathrm{ActEndstim\_a}+\frac{1-\mathrm{ActEndstim\_a}}{1+e^{\frac{\frac{-4\cdot \mathrm{ActEndstim\_b}}{\mathrm{ActEndstim\_c}}}{1-\mathrm{ActEndstim\_a}}\cdot([\mathrm{SUMACT\_END}]-\mathrm{ActEndstim\_c})}}$$

$$[\mathrm{SUMACT\_END}] = [\mathrm{MP}] + \mathrm{alfa11}\cdot[\mathrm{oxLDL}] + \mathrm{alfa12}\cdot[\mathrm{TINF}] + \mathrm{alfa13}\cdot[\mathrm{PLAQUE}]$$

$$\frac{d([\mathrm{PLAQUE}]\cdot V_{\mathrm{Endothelium}})}{dt} = +V_{\mathrm{Endothelium}}\cdot(\mathrm{Kdeg\_MP}\cdot[\mathrm{MP}]) + V_{\mathrm{Endothelium}}\cdot(\mathrm{Kdeg\_TINF}\cdot[\mathrm{TINF}]) - V_{\mathrm{Endothelium}}\cdot(\mathrm{KBcleanPLAQUE}\cdot[\mathrm{Bcells}]\cdot[\mathrm{PLAQUE}]) - V_{\mathrm{Endothelium}}\cdot(\mathrm{KRCT}\cdot[\mathrm{HDL}]\cdot[\mathrm{PLAQUE}]) + V_{\mathrm{Endothelium}}\cdot\left(\mathrm{ForFoamConstant}\cdot\left(\mathrm{ForFoamstim\_a}+\frac{1-\mathrm{ForFoamstim\_a}}{1+e^{\frac{\frac{-4\cdot \mathrm{ForFoamstim\_b}}{\mathrm{ForFoamstim\_c}}}{1-\mathrm{ForFoamstim\_a}}\cdot([\mathrm{SUMForFoam}]-\mathrm{ForFoamstim\_c})}}\right)\cdot\left(1-\mathrm{ForFoaminh\_a}-\frac{1-\mathrm{ForFoaminh\_a}}{1+e^{\frac{\frac{-4\cdot \mathrm{ForFoamstim\_b}}{\mathrm{ForFoaminh\_c}}}{1-\mathrm{ForFoaminh\_a}}\cdot([\mathrm{TAINF}]-\mathrm{ForFoaminh\_c})}}\right)\cdot[\mathrm{MP}]\cdot[\mathrm{oxLDL}]\right)$$

$$[\mathrm{SIGMOID\_ACTENDinh}] = \mathrm{ActEndinh\_a}+\frac{1-\mathrm{ActEndinh\_a}}{1+e^{\frac{\frac{-4\cdot \mathrm{ActEndinh\_b}}{\mathrm{ActEndinh\_c}}}{1-\mathrm{ActEndinh\_a}}\cdot([\mathrm{TAINF}]-\mathrm{ActEndinh\_c})}}$$

$$\frac{d([\mathrm{Bcells}]\cdot V_{\mathrm{Endothelium}})}{dt} = -V_{\mathrm{Endothelium}}\cdot(\mathrm{Kdeg\_Bcells}\cdot[\mathrm{Bcells}]) - V_{\mathrm{Endothelium}}\cdot(\mathrm{KBcleanoxLDL}\cdot[\mathrm{Bcells}]\cdot[\mathrm{oxLDL}]) - V_{\mathrm{Endothelium}}\cdot(\mathrm{KBcleanPLAQUE}\cdot[\mathrm{Bcells}]\cdot[\mathrm{PLAQUE}]) + V_{\mathrm{Endothelium}}\cdot\left(\mathrm{RecBcellsConstant}\cdot\left(\mathrm{RecBcellsstim\_a}+\frac{1-\mathrm{RecBcellsstim\_a}}{1+e^{\frac{\frac{-4\cdot \mathrm{RecBcellsstim\_b}}{\mathrm{RecBcellsstim\_c}}}{1-\mathrm{RecBcellsstim\_a}}\cdot([\mathrm{SUMRecBcells}]-\mathrm{RecBcellsstim\_c})}}\right)\right)$$

$$[\mathrm{SUMRecBcells}] = [\mathrm{MP}] + \mathrm{alfa8}\cdot[\mathrm{TAINF}] + \mathrm{alfa9}\cdot[\mathrm{ACT\_END}] + \mathrm{alfa10}\cdot[\mathrm{PLAQUE}]$$

$$[\mathrm{ACT\_END}] = \mathrm{ActEndConstant}\cdot[\mathrm{SIGMOID\_ACTENDstim}]\cdot(1-[\mathrm{SIGMOID\_ACTENDinh}])$$

$$[\mathrm{SUMoxidLDL}] = [\mathrm{MP}] + \mathrm{alfa1}\cdot[\mathrm{ACT\_END}]$$

$$[\mathrm{SUMForFoam}] = [\mathrm{MP}] + \mathrm{alfa2}\cdot[\mathrm{TINF}] + \mathrm{alfa3}\cdot[\mathrm{ACT\_END}]$$

$$[\mathrm{SUMRecTINF}] = [\mathrm{MP}] + \mathrm{alfa4}\cdot[\mathrm{TINF}] + \mathrm{alfa5}\cdot[\mathrm{ACT\_END}] + \mathrm{alfa6}\cdot[\mathrm{oxLDL}]$$

$$\frac{d([\mathrm{HDL}]\cdot V_{\mathrm{Endothelium}})}{dt} = -V_{\mathrm{Endothelium}}\cdot(\mathrm{KoutHDL}\cdot[\mathrm{HDL}]) + V_{\mathrm{Endothelium}}\cdot(\mathrm{KinHDL}\cdot[\mathrm{HDLC}]\cdot M) - V_{\mathrm{Endothelium}}\cdot(\mathrm{KRCT}\cdot[\mathrm{HDL}]\cdot[\mathrm{PLAQUE}])$$
